# Modulating Drug Release from Short Poly(ethylene glycol) Block Initiated Poly(L-lactide) Di-block Copolymers

**DOI:** 10.1007/s11095-022-03228-8

**Published:** 2022-04-26

**Authors:** Zein Azhari, Patricia Smith, Sean McMahon, Wenxin Wang, Ruth E. Cameron

**Affiliations:** 1 Department of Materials Science and Metallurgy, University of Cambridge, Cambridge, UK; 2 Ashland Specialties Ireland Ltd., National Science Park, Building V, Dublin Road, Petitswood, Mullingar, Co. Westmeath, Ireland; 3 The Charles Institute of Dermatology, School of Medicine and Medical Science, University College Dublin, Dublin, Ireland

**Keywords:** Drug Release, Processing, Hydrolysis, Bioresorbable, Biomaterials

## Abstract

This paper investigates drug release from a novel series of mPEG-functionalised PLLA polymers whose individual components (PEG and PLLA) have regulatory FDA approval. Two processing methods were explored to understand their effect on the morphology and drug release profiles of the polymers, with and without mPEG functionalisation. In the first method the polymer and Propranolol.HCl drug powders were mixed together before injection moulding. In the second method, supercritical CO_2_ was used to mix the polymer and drug before injection moulding. When non-functionalised PLLA was processed through injection moulding alone, there were no signs of polymer-drug interaction, and the drug was confined to crystals on the surface. This resulted in up to 85 wt% burst release of propranolol.HCl after one day of incubation. By contrast, injection moulding of mPEG-functionalised polymers resulted in the partial dissolution of drug in the polymer matrix and a smaller burst (50 wt% drug) followed by sustained release. This initial burst release was completely eliminated from the profile of mPEG-functionalised polymers processed via supercritical CO_2_. The addition of mPEG facilitated the distribution of the drug into the bulk matrix of the polymer. Paired with supercritical CO_2_ processing, the drug release profile showed a slow, sustained release throughout the 4 months of the study.

## Introduction

Bioresorbable polymers such as poly(*α*-hydroxyacids) have been extensively researched as drug delivery carriers to regulate and control the release of bioactive agents (1–3). Despite the perceived benefits of bioresorbable polymers as matrices for sustained drug release, the first phase of the drug release profile of poly(*α*-hydroxyacids) often displays an initial ‘burst’ which is characterised by a much higher concentration of drug released over a short length of time compared with the rest of the drug release profile (4, 5). The burst usually occurs as a result of drug dispersions at the surface of the polymer which readily dissolve into the surrounding medium (6, 7). This burst release phenomenon is often undesirable in drug delivery devices because it can lead to doses exceeding the toxic threshold and can hamper the overall sustained release therapy by creating a deficit in drug concentrations thereafter. Methods used to eliminate this effect tend to impact other factors such as drug loading, choice of polymer excipient and subsequent degradation (8).

If a fraction of the drug is well dispersed or dissolved in the polymer matrix, a second phase of drug release may be observed, displaying a prolonged period of sustained release whose kinetics may rely on several factors including: the rate of breakage of the ester bonds in the polyester, polymer swelling, pore formation and polymer-drug interactions (9). Finally, the third phase, if present, may be characterised by a steeper release of pharmaceutical products through the mass loss of bulk polymer fragments when the device has lost its mechanical integrity (10, 11).

The drug release profile from resorbable polyesters can be predominantly associated with the degradation of the polymer when drug is uniformly intermixed in the system. For example, the work of Hurrell *et al.* (12, 13) showed that, on incubation of drug-loaded polyglycolide (PGA), a sustained mode of drug release occurs once the polymers reach their critical molecular weight. At this point, a reaction erosion front is formed which progresses into the sample and induces the gradual release of the drug embedded within the polymer matrix.

Another factor which can affect drug release can include the level of crystallinity of a polymer. Active agents are excluded from the polymer crystal structure and are excluded to the amorphous regions of the polymer or at the surface (13). Miyajima *et al.* proposed that drug is excluded from polymer crystals and encouraged to crystallise in the amorphous areas of the polymer when they do not interact with the polymer matrix (14, 15). Drug and polymer miscibility could therefore ensure that the drug is homogeneously distributed in the polymer matrix. Supercritical CO_2_ has been used to blend polymers and drugs but can affect the structural integrity of the device through foaming once drug has been embedded into the polymer system. This processing technique can also be limited by the level of dissolution of drug and polymer (6).

Although little has been reported on the effect of mPEG functionalisation on release profiles, studies (16, 17) are reports of drug release from relatively high *M*_w_ PLLA-PEG di-block copolymers. Lee *et al.* (16) conducted swelling tests and drug release analysis of ibuprofen loaded PLLA-PEG di-block copolymers in the form of solvent cast films. The wt% content of PEG (*M*_w_ = 5000 Da) was varied from about 5 to 20 wt%, resulting in copolymers ranging in weight from 18 to 97 kDa. On incubation in PBS, they reported an increase in % water absorption with increasing PEG molar content, attributing this to increased material hydrophilicity with increased PEG monomer. Drug release mirrored the water sorption with films containing a higher wt% of PEG releasing ibuprofen more quickly. This was also confirmed by Zhu *et al.*. In contrast to Lee et al., who processed their materials by solvent casting, Zhu *et al.* processed their materials by compression moulding. Both gave an initial burst release in their drug release profiles which were only sustained for less than 50 days (17) and less than 20 days (16). The studies do not discuss the differences between release from a PLLA homopolymer compared with a mPEG-functionalised PLLA but suggest that drug release is initially mediated by water sorption into the polymer system and increases with increasing PEG content. Little is known about the effects of mPEG functionalisation and supercritical CO_2_ processing on the state of drug dispersion and consequent release.

This paper explores the potential application for drug release from a series of novel mPEG functionalised PLLA molecules previously reported by Azhari et al. (18). The study compares the effects of processing the polymers through injection moulding with and without supercritical CO_2_ (scCO2) assisted loading of the drug into the polymer matrix. The polymer series comprises high molecular weight mPEG functionalised PLLA polymers which have shown promise as a route to controlling rate and onset of degradation. The hydrolytic degradation rate was shown to depend only on the presence of mPEG and was little affected by mPEG length or PLLA length in the ranges studied.

This work investigates the impact of drug incorporation on polymer and drug morphologies and how this ultimately affects the drug release profile of the polymers. PEG end groups are explored with and without scCO2 drug incorporation to enhance drug/polymer miscibility. Both strategies are explored to enhance the distribution of drug in the polymer matrix, help reduce the initial burst release typically observed from such systems and pave the way for sustained release. When scCO_2_ is used, drug is incorporated in the polymer granules rather than in the final sample to maintain their shape.

## Materials and Methods

### Materials

The PLLA control, with an inherent viscosity of 1.0 dl/g was obtained from Purac (Gorinchem, Netherlands). mPEG functionalised PLLA’s were produced by Ashland and supplied from the Viatel™ bioresorbable polymer platform for this project. Propranolol.HCl was supplied from Sigma Aldrich. Liquid carbon dioxide was supplied by BOC gases.

### Synthesis of mPEG functionalised PLLA

mPEG functionalised PLLA with a mPEG *M*_*n*_ of 2000 g/mol (mPEG-PLLA) and constant PLLA *M*_*n*_s approaching 80 kg/mol were synthesised by mPEG initiation through the ring opening polymerisation (ROP) of L-lactide as described in further detail in Azhari et al. (18). Briefly, polymerization of the monomer L-lactide was conducted via ring-opening-polymerization in the presence of catalyst Tin(II) ethyl hexanoate. The reaction was initiated by a hydroxyl-functionalised methoxy-terminated PEG (mPEG). This reaction yielded long chains of poly L-lactide (PLLA) connected to an mPEG terminal end group.

Two methods of drug loading were investigated and propranolol.HCl was selected for the drug release studies because it is known to be thermally stable, is soluble in PBS and detectable in the UV range. The first method involved drug loading of propranolol hydrochloride (HCl) into the polymer via direct injection moulding. The second method of drug incorporation was achieved via supercritical CO2 loading of as-synthesised mPEG-PLLA granules before injection moulding. The two methods are described below.

#### Drug Loading

##### Method 1: Propranolol.HCl mixed with polymer granules during injection moulding

A nominal 5 wt% drug loading in all polymers was achieved by separately weighing the drug and polymer, supplied in solid form, using a Sartorius CP124S with d = 0.1 mg. The two were crudely mixed in anti-static weighing boats before injection moulding. Nominal wt% drug loading is described in Eq. 1.

$$Weight\% Drug\ Loading=\frac{M_{drug}}{M_{polymer.}}$$

##### Method 2: Propranolol.HCl mixed with polymer granules during supercritical CO_2_ processing

mPEG-PLLA was finely ground to form a powder state which was then added to the stainless-steel autoclave with the equivalent of 5 wt% of propranolol.HCL. The autoclave was heated to 145°C while CO2 was pressurized to 180 Bar. This resulted in a supercritical CO^2^ state with density of 302.99 kg/m3 which assisted with plasticizing the polymer for improved drug compounding. The autoclave was then stirred at 150 RPM for 2 h at these conditions to complete compounding of the drug within the polymer. Precautions were taken to minimize moisture to avoid hydrolytic degradation.

In both cases, the different blends of polymer and propranolol.HCl were micro-injection moulded (DSM Research, X’plore) under ambient temperature. A custom made dumbbell mould with a 5 mL cavity was used. Three stages of injection including injecting, filling and holding were set to pressures of 9 bar, 5 bar and 5 bar. The processing temperatures were adjusted to the minimum temperature, ranging from 185 to 245*°*C, found to result in visually uniform specimens while the mould temperature was kept at ambient temperature. The resulting dumbbell specimens were cut into identical cuboids with dimensions of 4 × 4 × 2 mm using an Accustom-5 (Struers) blade.

### Differential Scanning Calorimetry (DSC)

Differential Scanning Calorimetry (DSC) (Q100, TA Instruments) was used. The baseline, subtracted from all subsequent measurements, was determined by running an empty aluminium pan over the instrument temperature range of −90*°*C to 550*°*C. For the calibration of heat capacity, two sapphire standards were used as reference and sample. Enthalpy and temperature were calibrated with indium. Testing was carried out in triplicate on polymer samples ranging between 2 and 10 mg which were sealed in aluminium pans and heated over a range of −80*°*C to 250*°*C at a rate of 20*°*C/min. A nitrogen flow rate of 50 mL/min was maintained throughout the scans. The TA Instruments software package (Universal Analysis) was used to determine the glass transition temperature *T*_g_, taken at the inflection point while the crystallisation temperature, *T*_c_, and melting temperature, *T*_m_, were respectively recorded at the exothermic and endothermic peaks. The latent heat of fusion, ∆*H*_m_, was estimated from the area under the endothermic peak. The degree of crystallinity was estimated based on Eq. 2, below:

$$Degree\ of\ Crystallinity=\frac{\Delta {H}_m}{\Delta {H^0}_m}$$

A value of 143 J.g^*−*1^ (∆H^0^_m_) was used for 100% crystalline PLLA (19).

### Fourier Transform Infrared Spectroscopy (FTIR)

FTIR spectra were obtained on a Bruker (Tensor 27) spectrometer between 520 cm^*−*1^ and 4000 cm^*−*1^ in transmission mode. The resolution was 4 cm^*−*1^. Peak wavelengths were identified using the Omnic software.

### Wide Angle X-Ray Scattering (WAXS)

Wide Angle X-Ray Scattering (WAXS) was carried out using a Philips X^*1*^Pert PW1830 generator with an incident beam of Cu K*α* radiation (1.540598). The diffraction patterns were acquired in a 2*θ* angle range of 2 - 50^*°*^with a step size of 0.050^*°*^at a scanning speed of 0.020^*°*^/s.

### Scanning Electron Microscopy (SEM)

Scanning Electron Microscopy (SEM) was carried out using a JEOL 5800 scanning electron microscope. Fracture surfaces were prepared by indenting the edge of the injection moulded sample and splitting the material at the crack location. Polymer samples were coated with palladium. An accelerating voltage of 5 eV was used to avoid sample charging and cracking.

### Lactate Assay

Lactate concentration was determined by an enzymatic assay kit obtained from Sigma Aldrich, which results in a colorimetric (570 nm) product, proportional to the lactate present. A standard curve was generated with known amounts of 0, 2, 4, 8 and 10 μL lactate (lactate probe, provided in the kit), and used to determine the lactate content in the samples. The lactate assay buffer was allowed to come to room temperature before use. The lactate enzyme mix was reconstituted in 220 mL of lactate assay buffer and thoroughly mixed by pipetting. A master mixture made up of 46 μL of lactate buffer, 2 μL of the reconstituted lactate enzyme mix and 2 μL of the lactate probe. 50 μL of the master mixture was added to each well in a 96 well plate containing 50 μL of the 1/200 diluted PBS solutions surrounding the degrading materials. A calorimetric reading was recorded at 570 nm on a Spectrostar microplate reader (BMG, Labtech). Lactate concentration in the PBS surrounding each polymer was determined by using a value of 89.07 Da for the *M*_w_ of lactate as specified in the lactate assay kit from Sigma Aldrich.

### Drug Release Measurements

**Figure 1:**
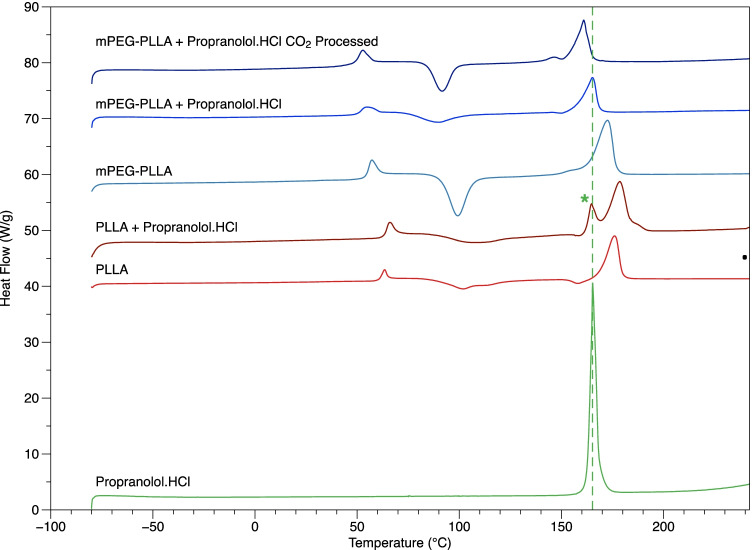
DSC thermograms of Propranolol.HCl, neat PLLA, neat mPEG-PLLA polymer, PLLA and mPEG-PLLA polymers loaded with Propranolol.HCl through mixing before injection moulding and mPEG-PLLA polymer loaded with Propranolol.HCl through mixing during scCO_2_ processing. The dotted line indicates the position of the melting peak of Propranolol.HCl and the asterix points to the presence of the melting peak in the PLLA polymer. The thermograms are offset for clarity.

Six drug loaded samples were processed for each of the polymers listed in Fig. 1. The samples were individually incubated in 7 mL bijou tubes containing 0.01 M PBS at pH = 7.4 with a material to PBS ratio of 6 mg to 1 mL which corresponded to approximately 3 mL of PBS. The tubes were placed in an incubator set at 37*°*C. The absorbance of propranolol.HCl at 289 nm was monitored by UV-VIS spectroscopy. A standard curve was generated with known amounts of propranolol.HCl, and used to determine the amount of drug released into the surrounding medium. Measurements were initially taken every 2 h, twice a week, once a week and down to once every two weeks as the amount of propranolol.HCl released decreased over time. The PBS medium was replaced by a new PBS blank after each measurement and the UV-ViS cuvette was rinsed with PBS solution in between measurements.

## Results

### Differential Scanning Calorimetry (DSC)

Figure 1 shows the DSC thermogram of propranolol.HCl and 5 wt% drug-loaded PLLA, mPEG-PLLA and scCO_2_ processed mPEG-PLLA. The trends observed in Fig. 1 and the values listed in Table I suggest that on addition of mPEG to neat PLLA, the T_g_ value decreases with negligible changes observed on incorporation of propranolol.HCl. The melting temperatures of mPEG-PLLA polymers decrease on addition of propranolol.HCl. Both drug loaded PLLA and scCO2 processed mPEG-PLLA show two melting peaks.

**Table I:** Thermal properties of propranolol.HCl, processed and drug-loaded PLLA and mPEG-PLLA

	T_g_ (*°*C)	T_m_ (*°*C)
Propranolol.HCl*	36.1 ± 0.1	163.3 ± 0.17
PLLA	61.7 ± 0.4	175.7 ± 0.9
mPEG-PLLA	55.5 ± 0.2	172.4 ± 1.0
PLLA + Propranolol.HCl	64.8 ± 0.2	164.4 ± 0.5**
mPEG-PLLA + Propranolol.HCl	52.0 ± 0.5	167.0 ± 1.7
mPEG-PLLA + Propranolol HCl CO_2_ Processed	51.9 ± 0.9	147.0 ± 1.2**

*Thermal transition transitions derived from second heating curve as this is more representative of the conditions which the drug will be subjected to once processed into the polymer samples.

**A second melting peak was observed for drug loaded PLLA and scCO2 processed mPEG-PLLA: 178.2 ± 0.5*°*C and 161.9 ± 0.9*°*C.

### Fourier Transform Infrared Spectroscopy (FTIR)

**Figure 2:**
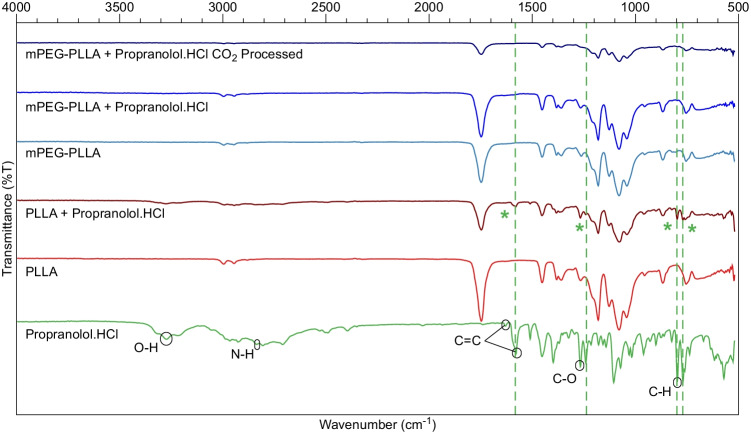
FTIR spectra of Propranolol.HCl, neat PLLA, neat mPEG-PLLA polymer, PLLA and mPEG-PLLA polymers loaded with Propranolol.HCl through mixing before injection moulding and mPEG-PLLA polymer loaded with Propranolol.HCl through mixing during CO_2_ processing. The dotted lines indicate the characteristic peaks ascribed to Propranolol.HCl and the asterix points to the presence of these peaks in the PLLA polymer. The spectra are offset for clarity.

FTIR was used to determine the level of interaction between the drug and polymer across the two drug-loading techniques, injection moulding and supercritical CO_2_ loading. Differences in the FTIR spectra are observed between pure and 5 wt% drug-loaded PLLA, mPEG-PLLA polymers and the scCO_2_ processed mPEG-PLLA polymer shown in Fig. 2. The pure forms of PLLA and mPEG-PLLA were identical except for a peak at approximately 800 cm^−1^. The peak is not resolved in the drug loaded mPEG-polymers. Furthermore, the drug loaded PLLA spectra displayed multiple peaks corresponding to those identified in propranolol.HCl while there were no characteristic peaks assigned to the drug in both the drug-loaded mPEG-PLLA spectra.

### Wide Angle X-Ray Scattering (WAXS)

**Figure 3:**
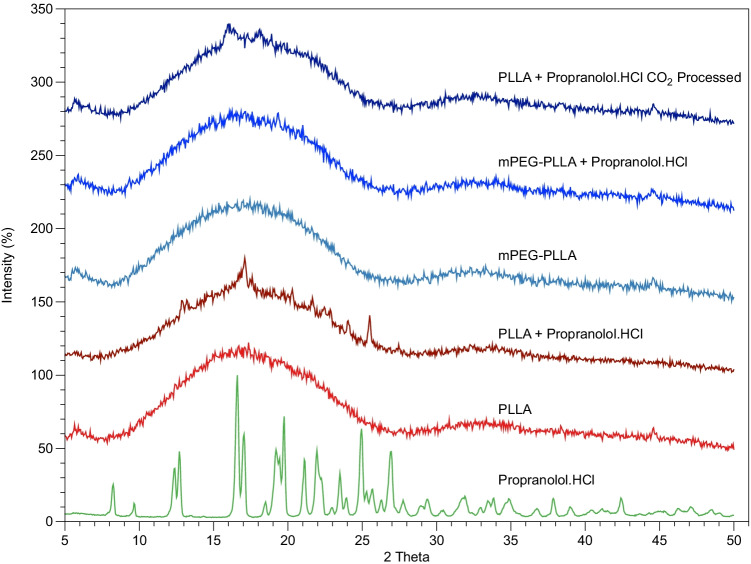
WAXS traces of Propranolol.HCl, neat PLLA, neat mPEG-PLLA polymer, PLLA and mPEG-PLLA polymers loaded with Propranolol.HCl through mixing before injection moulding and mPEG-PLLA polymer loaded with Propranolol.HCl through mixing during scCO_2_ processing. The traces are offset for clarity.

Figure 3 shows the XRD patterns acquired on the 5 wt% drug-loaded PLLA, mPEG-PLLA and scCO_2_ processed mPEG-PLLA pre drug-release while Fig. 3 shows them post drug- release. After processing, sharp peaks, characteristic of propranolol.HCl, are only observed in the drug-loaded PLLA polymer. Broader peaks can be distinguished in the scCO2 mPEG-PLLA polymer.

### Scanning Electron Microscopy (SEM)

**Figure 4:**
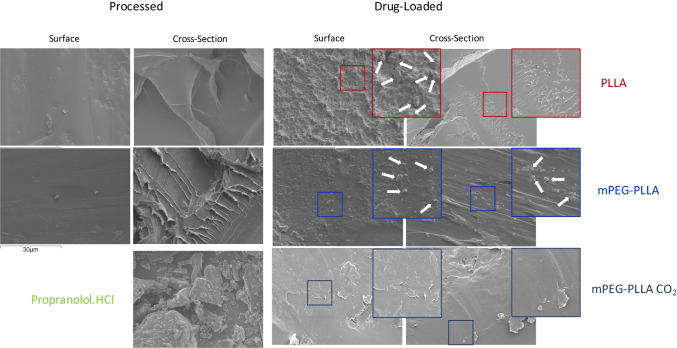
SEM images of (Processed, on the left) Propranolol.HCl, neat PLLA, neat mPEG-PLLA polymers and (Drug-Loaded, on the right) PLLA and mPEG-PLLA polymers loaded with Propranolol.HCl through mixing before injection moulding and mPEG-PLLA polymer loaded with Propranolol.HCl through mixing during CO_2_ processing. The arrows point to possible crystalline drug granules due to the likeness to the granules in the Propranolol.HCl image.

Figure 4 shows an SEM micrograph for propranolol.HCl, sample surfaces and fracture surfaces of pure PLLA and PLLA-PEG 2000 and sample surfaces and fracture surfaces of 5 wt% drug-loaded PLLA, 5 wt% drug-loaded mPEG-PLLA and scCO_2_ processed mPEG-PLLA polymers at a magnification of × 2000. Differences in morphology, drug incorporation and miscibility were examined for fracture and sample surfaces. Drug-loaded PLLA displays a very rough surface morphology relative to the pure PLLA surface morphology. This roughness is much less prominent in the mPEG-PLLA polymers.

### Lactate Assay

**Figure 5:**
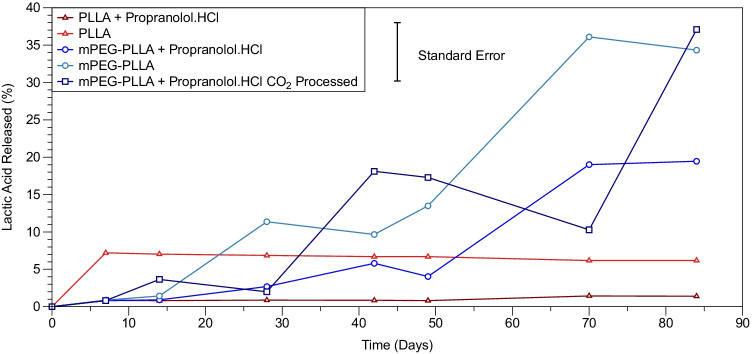
Lactic Acid release curves of PLLA and mPEG-PLLA polymers loaded with Propranolol.HCl through mixing before injection moulding and mPEG-PLLA polymer loaded with Propranolol.HCl through mixing during CO_2_ processing. The error bar represents the standard error in 6 measurements for a representative sample condition.

Figure 5 shows cumulative lactate concentration as a function of time in pure PLLA and mPEG-PLLA and 5 wt% drug-loaded PLLA, 5 wt% drug-loaded mPEG-PLLA and scCO2 processed mPEG-PLLA polymers. PLLA displays minimal to no release over the duration of the study. Upon incorporation of PEG in the mPEG-PLLA polymers, soluble lactic acid is released from the onset of the study.

### Drug Release Curves

**Figure 6:**
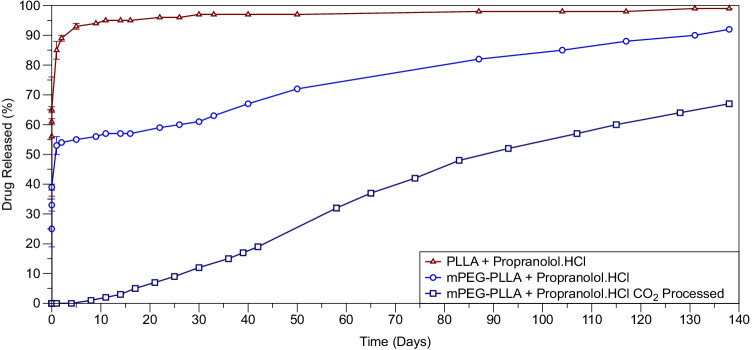
Drug release curves of PLLA and mPEG-PLLA polymers loaded with Propranolol.HCl through mixing before injection moulding and mPEG-PLLA polymer loaded with Propranolol.HCl through mixing during CO_2_ processing. The error bars represent standard errors calculated on six measurements.

Figure 6 shows cumulative drug concentration as a function of time in the 5 wt% drug-loaded PLLA, mPEG-PLLA and scCO2 processed mPEG-PLLA polymers. The PLLA and mPEG-PLLA polymers display a level of burst release in the initial 48 h of the study followed by a plateau or very little release for PLLA and a sustained release over the duration of the study for the PEG-functionalised PLLA polymer. In contrast, scCO2 mPEG-PLLA displays a sustained release from the start of the study.

## Discussion

### The effects of processing and mPEG on the distribution and morphology of the drug within the polymer

Drug release is significantly affected by the morphology of the drug in the polymer. Surface drug crystals contribute to burst release, while a drug present in an amorphous state, dispersed within the bulk, is more likely to be released in a slow and sustained mode (20–23). Furthermore, in comparison to an amorphous drug, a crystalline drug presents long range order, so additional energy is required to break the bonds during the dissolution process leading to less favourable bioavailability (24).

In Fig. 4, the drug-loaded PLLA polymer displays a rough surface morphology relative to the pure PLLA surface morphology which may reflect a solid dispersion of propranolol.HCl crystals. It is not clear if drug crystals are incorporated into the polymer fracture surface which may suggest that the drug is present as an amorphous dispersion in the matrix. However, on analysis of the DSC thermogram of neat PLLA (Fig. 1), a crystallinity of 17.1% was estimated in the polymer matrix and a crystallinity of 29.2% was estimated in PLLA loaded with drug. The crystallinity in the polymer matrix may have led to drug being excluded from polymer crystals and encouraged to crystallise in the amorphous regions or at the surface of the polymer as proposed by Miyajima *et al.* (14, 15). Furthermore, the propranolol.HCl melting peak in the DSC thermogram (Fig. 1) of drug-loaded PLLA, and the characteristic crystal peaks in the XRD spectrum (Fig. 3), confirm that the drug is present in a crystalline state within the sample. Crystalline drug confined to the surface of PLLA would contribute to a mode of instant burst release via dissolution (4, 20).

The surface morphology in the mPEG-PLLA polymer, prepared by mixing propranolol.HCl with polymer granules before injection moulding, shows lower surface roughness than the PLLA sample which may suggest that the polymer is more intermixed with the drug when mPEG is present. Drug crystals can be observed on the fracture surface (Fig. 4) suggesting that the drug is incorporated into the bulk polymer matrix. PEG is widely used in pharmaceutical formulations because it can contribute to drug solubility (20, 25) which may explain why the surfaces in SEM are smoother. However, XRD suggests that some of the drug is also in a crystalline state (Fig. 3).

mPEG-PLLA scCO2, prepared by mixing propranolol.HCl and polymer granules during supercritical CO_2_ processing, does not exhibit any form of roughness or particles in the SEM images (Fig. 4). The XRD spectrum of the processed material (Fig. 3) also shows a near amorphous morphology with the absence of characteristic propranolol.HCl peaks. In combination, these results suggest that the drug is amorphously dispersed within the polymer. These observations are in line with Morgan *et al.* (26) who observed the same characteristic propranolol.HCl sharp edge particles in their SEM images. Upon investigation of the morphology of hot melt extruded 10 wt% propranolol.HCl loaded methyl methacrylate Eudragit copolymers, they observed smooth surface morphologies proposing that the drug is present in the polymer matrix in the form of an amorphous solid dispersion. They also support these findings with XRD which did not exhibit any characteristic propranolol.HCl peaks.

**Figure 7:**
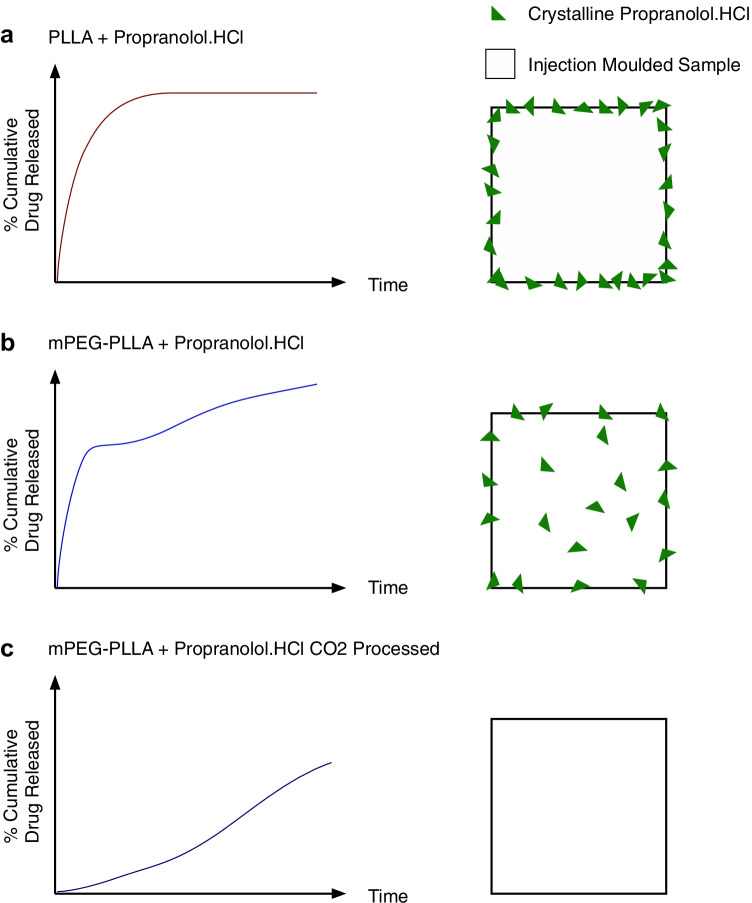
Summary diagram of the effect of drug loading (through mixing propranolol.HCl and polymer before injection moulding or during supercritical CO_2_ processing) and polymer processing (through incorporation of mPEG) on the mode of drug release. (a) When PLLA granules and propranolol.HCl were mixed before injection moulding, propranolol.HCl crystallised at the polymer surface resulting in a burst release. (b) When mPEG-PLLA granules and propranolol.HCl were mixed before injection moulding, a limited amount of propranolol.HCl crystallised at the polymer surface and in the polymer matrix contributing to a burst release. The rest of the drug was dissolved in the polymer as an amorphous dispersion resulting in a sustained release controlled by gradual polymer degradation. (c) When mPEG-PLLA granules and propranolol.HCl were mixed during supercritical CO_2_ processing, the drug was completely dissolved in the polymer matrix resulting in a sustained release controlled by gradual polymer degradation.

As summarised in Fig. 7, these results suggest that Propranolol.HCl is present as a crystal dispersion at the surface of the PLLA control while these formations are less evident in the mPEG-functionalised polymers. In mPEG functionalised polymers processed with scCO2, the drug is exclusively amorphous.

### The effects of processing and mPEG on drug- polymer interaction

Both the morphology of the drug and its interaction with the polymer are likely to affect drug release and distribution (20, 21, 25).

FTIR may be used to indicate the level of intermolecular interaction between a polymer and a drug. The appearance of additional peaks other than those assigned to the studied materials or the shifting or broadening of peaks could suggest that some interaction is occurring between the two substances (27, 28). The presence of peaks belonging to propranolol.HCl and PLLA confirm that no interaction is occurring in the PLLA control (Fig. 2).

The pure forms of PLLA and mPEG-PLLA were identical except for a peak at approximately 800 cm^*−*1^, linked to out of plane C-H hydrogen bonding in crystalline PEG, in the mPEG-functionalised polymer. While no solid conclusions can be made based on such a weak intensity peak, the disappearance of the peak at 800 cm^*−*1^ for the two mPEG-PLLA drug-loaded polymers, prepared by mixing polymer granules and propranolol.HCl before injection moulding and during supercritical CO_2_ processing, are suggestive of a transition to an amorphous morphology of mPEG (29–31). PEG has been shown to improve drug dissolution (32). However, there were no characteristic peaks assigned to the drug in both the drug-loaded mPEG-PLLA spectra which may indicate that the peaks are masked by the characteristic polymer peaks. There are also no characteristic O-H bands between 3000 and 4000 cm^*−*1^ assigned to intra-molecular hydrogen bonding.

In Fig. 1, two *T*_m_ values are observed in PLLA at 164.4°C and 178.2°C representing the individual polymer and drug melting temperatures and strongly suggesting that no interaction is occurring between the two. The *T*_g_ in PLLA shows a shift from 61.7°C to 64.8°C which may indicate that the drug is reducing confirmational flexibility. In comparison, mPEG-PLLA displays a single *T*_m_ value shifted from 172.4°C to 167°C intermediate to the individual *T*_m_ values of the polymer and drug (165.1°C). The main *T*_m_ of mPEG-PLLA CO_2_ shifts to 161.9*°*C and a smaller peak appears at 147*°*C. Although both polymers were relatively amorphous, the percentage crystallinity in mPEG-PLLA (18.7%), prepared by mixing the drug with polymer granules before injection moulding, was greater than mPEG-PLLA CO_2_ (6.3%) suggesting that a higher proportion of the drug is present in an amorphous state in the latter polymer. Furthermore, the *T*_g_ for the two mPEG-PLLA drug-loaded polymers shifted from 55.5*°*C (mPEG-PLLA) to 52.0*°*C for mPEG-PLLA loaded with drug before injection moulding and to 51.9*°*C for mPEG-PLLA CO_2_. Both values for drug loaded polymers shifted closer to the 36.1*°*C *T*_g_ for propranolol.HCl (derived from the 2nd heating curve) suggesting a degree of miscibility between the drug and the polymer. These findings are in contradiction with work by Takka *et al.* (33) who suggest that an increase in Tg is related to a greater polymer-drug interaction while a decrease in *T*_g_ indicates that the polymer only acts as a plasticiser with no further interaction with the polymer matrix.

The data suggest that mixing PLLA granules with propranolol.HCl before injection moulding does little to encourage polymer/drug miscibility. Incorporating mPEG in the polymer may encourage drug dissolution in the matrix and processing the polymer with supercritical CO_2_ has shown a greater proportion of the drug present in an amorphous state.

### The effects of processing and mPEG on drug release profiles

The drug release mechanism from a degrading polymer is often triphasic (34). The first phase is generally attributed to a mode of burst release occurring in the first few hours, the second phase is attributed to the diffusion of drug through the polymer matrix, existing pores and the gradual solubilisation of the polymer. The final phase is primarily attributed to the release of drug as a result of polymer breakdown and may also show either an increase in drug release rates or a decrease in release rates due to a depletion of the drug loaded into the matrix.

Drug crystals contribute to a mode of burst release when located at the surface of the polymeric device, they tend to dissolve away over a short timescale (35, 36). The drug morphology varied *vis- a-vis* the different polymers in the series, PLLA tended to contain drug crystals at the surface while the inclusion of mPEG seemed to display a more intermixed distribution of solid drug dispersion in the bulk of the polymer with some roughness also observed at the surface (Fig. 4). This difference in drug crystal distribution may explain why most drug is released via a burst mode in PLLA while a burst release mode, also seen in the mPEG-PLLA polymer, is followed by a secondary sustained release (Fig. 6).

Zeng *et al.* (37) examined the effects of drug dissolution on the burst release of electrospun PLLA polymers. They observed doxorubicin hydrochloride drug crystals segregated to the surface owing to the lack of solubility between the solvent, PLLA and the drug, they successfully eliminated this burst effect after treating the doxorubicin hydrochloride with ammonia making the drug lipophilic and able to dissolve into PLLA. In a separate study, a layer by layer polyelectrolyte coating approach has been shown to be a good tactic for limiting burst release from polymeric devices as a measure of covering the drug particles present on the surface (38). Fig. 6 indicates that processing the polymers via supercritical CO_2_ completely eliminates the burst release, this may be because there were no indications of the drug being crystalline as well as the possibility of some level of polymer and drug interaction.

The second phase of drug release involves the diffusion of drug through the polymer matrix alongside the solubilisation of the polymer matrix associated with lactic acid release. A plateau is observed for the PLLA polymer just below 100% indicating that no drug is being released further to the initial burst release (Fig. 6).

Throughout the duration of the drug release study, indications of polymer solubilisation are not observed in PLLA (not supercritical CO_2_ processed) as confirmed by the absence of lactic acid release in Fig. 5. The addition of mPEG results in the formation of a slope or ‘S’ shape in the mPEG-PLLA-PEG polymer indicating the onset of mass loss (Fig. 6) corresponding to the onset of lactic acid release (Fig. 5). Processing the polymer via supercritical CO_2_ before injection moulding has the effect of imparting a slow sustained release in the polymer with no observed plateau or changes to the rate of drug release. These findings are summarised in Fig. 7.

The final phase of drug release is generally ascribed to the breakdown of the polymer matrix encapsulating the drug particles. At this point in the study, no drug has been released in PLLA (Fig. 6), due to the burst release of the drug present on the surface and the absence of any polymer degradation (18). The addition of mPEG to PLLA shows an inflection in the drug release graph at around 20 days which approximately correlates with the onset of lactic acid release (Fig. 5). Processing the polymer via supercritical CO_2_ results in a more linear, sustained release than the polymer processed by mixing the drug with polymer through direct injection moulding. This is owed to a greater interaction at a molecular level between the polymer and drug. The release of lactic acid is also consistent for this polymer.

As summarised in Fig. 7, injection moulding the drug with non-functionalised PLLA led to drug crystals being confined to the surface of the polymer, resulting in burst release. mPEG functionalisation of the polymer contributed to more of the drug being dispersed in the matrix which translated to a burst release for drug present as crystals at the surface of the polymer, followed by a sustained release governed by polymer degradation for drug dissolved in the polymer matrix. Finally, processing mPEG functionalised polymer and the drug via supercritical CO_2_ resulted in visibly uniform samples with uniformly dispersed amorphous drug in the polymer matrix, completely eliminating the initial burst release and showing a sustained mode of release which began at the onset of lactic acid release.

## Conclusion

The findings presented in this paper highlight the potential of mPEG functionalisation of PLLA coupled with scCO2 impregnation of API to achieve sustained and predictable drug release profiles throughout the lifetime of a medical device sustained release depot. In PLLA, it was found that the initial burst effect was the dominant mechanism of drug release accounting for approximately 85 wt% of propranolol.HCl release profile without further observations throughout the duration of the study. Once mPEG was included into the polymer, further release was seen in all polymers after a smaller amount of drug was released through a burst phase (approximately 50 wt% after 1 day of incubation). Processing the mPEG functionalised polymer via supercritical CO_2_ eliminated the initial burst release entirely due to the amorphous distribution of the drug in the matrix.

## Abbreviations

CO_2_: Carbon dioxide
DSC: Differential Scanning Calorimetry
FTIR: Fourier Transform Infrared Spectroscopy
FDA: Food and Drug Administration
HCl: Hydrochloride
RPM: Revolutions per minute
PBS: Phosphate buffered saline
PEG: Poly(ethylene glycol)
mPEG: Methoxy-Poly(ethylene glycol)
PLLA: Poly(L-lactide)
SEM: Scanning Electron Microscopy
scCO_2_: Supercritical CO_2_
UV-ViS: Ultra-Violet Visible Spetroscopy
WAXS: Wide Angle X-Ray Scattering
